# Comparative transcriptomic analysis reveals region-specific expression patterns in different beef cuts

**DOI:** 10.1186/s12864-022-08527-3

**Published:** 2022-05-20

**Authors:** Tianliu Zhang, Tianzhen Wang, Qunhao Niu, Xu Zheng, Haipeng Li, Xue Gao, Yan Chen, Huijiang Gao, Lupei Zhang, George E. Liu, Junya Li, Lingyang Xu

**Affiliations:** 1 Institute of Animal Science, Chinese Academy of Agricultural Sciences, Yuanmingyuan West Road 2#, Haidian District, Beijing, 100193 China; 2 Animal Genomics and Improvement Laboratory, United States Department of Agriculture-Agricultural Research Services, Beltsville, MD 20705 USA

**Keywords:** Meat quality, Region-specific expressed gene, Co-expression, Region-specific module, Beef cut, Cattle

## Abstract

**Background:**

Beef cuts in different regions of the carcass have different meat quality due to their distinct physiological function. The objective of this study was to characterize the region-specific expression differences using comparative transcriptomics analysis among five representative beef cuts (tenderloin, longissimus lumborum, rump, neck, chuck).

**Results:**

We obtained 15,701 expressed genes in 30 muscle samples across five regions from carcass meat. We identified a total of 80 region-specific genes (RSGs), ranging from three (identified in the rump cut) to thirty (identified in the longissimus lumborum cut), and detected 25 transcription factors (TFs) for RSGs. Using a co-expression network analysis, we detected seven region-specific modules, including three positively correlated modules and four negatively correlated modules. We finally obtained 91 candidate genes related to meat quality, and the functional enrichment analyses showed that these genes were mainly involved in muscle fiber structure (e.g., *TNNI1*, *TNNT1*), fatty acids (e.g., *SCD*, *LPL*), amino acids (*ALDH2*, *IVD*, *ACADS*), ion channel binding (*PHPT1*, *SNTA1*, *SUMO1*, *CNBP*), protein processing (e.g., *CDC37*, *GAPDH*, *NRBP1*), as well as energy production and conversion (e.g., *ATP8*, *COX8B*, *NDUFB6*). Moreover, four candidate genes (*ALDH2*, *CANX*, *IVD*, *PHPT1*) were validated using RT-qPCR analyses which further supported our RNA-seq results.

**Conclusions:**

Our results provide valuable insights into understanding the transcriptome regulation of meat quality in different beef cuts, and these findings may further help to improve the selection for health-beneficial meat in beef cattle.

**Supplementary Information:**

The online version contains supplementary material available at 10.1186/s12864-022-08527-3.

## Background

Beef, as a source of human food, contributes to an important part of a healthy diet and contains essential nutritional components (e.g., protein, polyunsaturated fatty acids, essential amino acids, minerals) [1, 2]. Improving the nutritional value of beef has received considerable attention as the increasing of people’s consumption level in recent years [3]. Beef carcass meat can be divided into retail cuts, such as tenderloin (psoas major), longissimus lumborum (striploin), rib, brisket, topside, shank, neck, and rump [1]. These beef cuts at different regions of the carcass meat have their natural physiological function, which can affect meat quality. Beef cuts from diverse regions may have different muscle fiber types and distributions [4], chemical and fatty acid profiles [5], and metabolic patterns, resulting in specific meat quality properties and sensory characteristics [6]. A higher proportion of fiber number, area percentages, and density of type IIA and IIB were found in tenderloin and longissimus lumborum [7]. Meanwhile, a higher percentage of type I fiber was found in tenderloin, which results in a lower fat content and tenderness for meat [7]. Moreover, tenderloin showed an obvious change from high to low in mitochondrial concentration and mitochondrial oxygen consumption with the increasing storage days when compared to longissimus lumborum [8].

High-throughput transcriptomics provides a more sensitive and precise analytical approach to comprehensively explore transcriptional landscapes in biological systems [9]. Recently, several studies have been performed to investigate the molecular basis of the meat quality in cattle using transcriptomic approaches. Fonseca et al. explored global gene expression differences in various beef cuts, and they found several potential candidate genes related to meat tenderness [10]. Yu et al. explored the muscle-specific molecular differences between the tenderloin and longissimus lumborum in the early postmortem period of cattle, and their results revealed 65 differentially expressed genes related to energy production and conversion, transcription, oxidative phosphorylation [4]. Meng et al. reported on the difference in meat quality between Simmental and Chinese native cattle in longissimus lumborum, and identified two important signaling pathways closely linked to meat quality, including endoplasmic reticulum and adipocytokine signaling pathway, and identified several candidate genes (*LEPR*, *HSPA12A,* and *CAPN1*) [11]. However, elucidating the potential mechanisms of expression regulatory differences related to meat quality among different beef cuts have not yet been explored.

To investigate the effect of different beef cuts on meat quality, we performed a comparative transcriptomic analysis on five types of beef cuts from adult Chinese Simmental beef cattle. In this study, we first evaluated the expression level of candidate genes in 30 muscle samples across five different regions. We identified region-specific genes (RSGs) related to the distinct meat quality of beef cuts and detected regulatory transcription factors (TFs) for RSGs. Then, we assessed the correlation between gene expression levels in different beef cuts, and obtained region-specific modules. Finally, we identified several important candidate genes showing diverse expression patterns among beef cuts and found these genes were differentially expressed, which contribute to meat quality with essential nutritional components.

## Results

### RNA sequencing and transcriptomes analysis

**Fig. 1:**
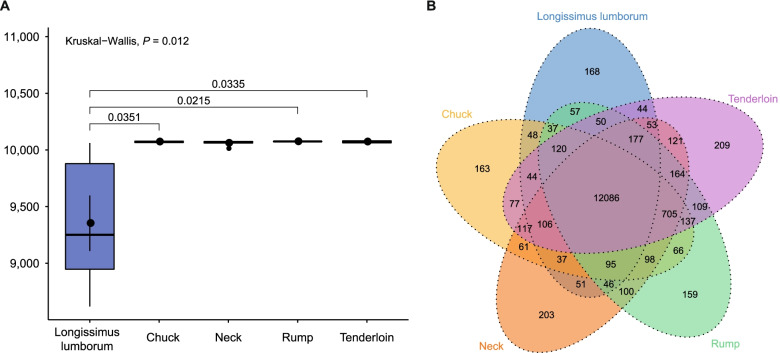
Expressed gene numbers, shared and unique genes in cattle longissimus lumborum, chuck, neck, rump, and tenderloin cuts. **a** The number of expressed genes in five types of beef cuts. The number of expressed genes are significantly different among beef cuts. (*P* < 0.012). **b** Number of shared and unique expressed genes identified in five types of beef cuts

A total of 682,547,111 raw paired-end reads (204.77 Gb) were generated from RNA sequencing of beef cut samples. Specifically, an average of 22.75, 23.61, 23.26, 22.11, and 22.02 million reads were obtained from the tenderloin, longissimus lumborum, rump, neck, and chuck cuts, respectively (Additional file 1: Table S1). In total, 96.97% of the raw reads passed the quality control, and an average of 95.27% (ranging from 94.23 to 97.02%) clean reads were mapped to the bovine reference genome ARS-UCD1.2. To quantify the gene expression level, the FPKM values of genes were calculated based on the length of the gene and the read counts mapped to the gene, and 15,701 genes were identified using StringTie software [12]. In this study, we found the total number of expressed genes was significantly lower in longissimus lumborum (9352 ± 600) than those in the chuck (10,071 ± 8), neck (10,060 ± 24), rump (10,074 ± 4), and tenderloin cuts (10,071 ± 11) (*P* < 0.05 was considered as the significant level) (Fig. 1a, Additional file 1: Table S1). In addition, 12,086 genes were commonly expressed in five beef cuts, and 209, 168, 159, 203, and 163 genes were uniquely expressed in tenderloin, longissimus lumborum, rump, neck, and chuck cuts, respectively (Fig. 1b). To eliminate the influence of confounding factors at the experimental level, we retained the genes with FPKM values greater than one in the six biological replicate samples. A total of 4511 genes were obtained for the downstream analyses.

### Region-specific expression patterns analysis

**Fig. 2:**
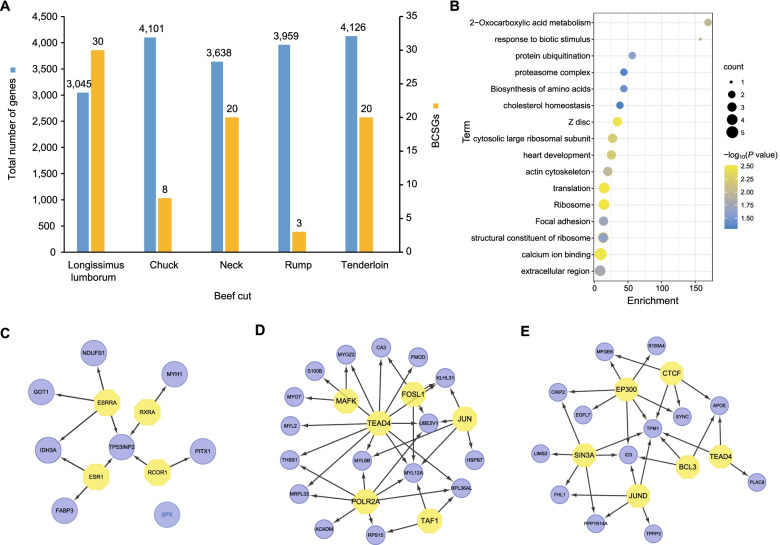
RSGs identification and functional analysis. **a** Distribution of the number of genes and RSGs in five types of beef cut. **b** Functional annotation and enrichment distribution of RSGs. The x-axis represents enrichment; the y-axis represents the function term. Color intensity represents the significance of the RSG; yellow represents highly expressed and blue represents lowly expressed. **c**, **d**, **e** TF analysis of RSGs in chuck, tenderloin, and neck cut, respectively. Yellow nodes represent TFs and blue nodes represent regulatory target genes

Based on the similar detection methods as described by a previous study [13], we identified a total of 80 RSGs in 4511 genes (ranging from 3045 to 4126) from five types of beef cuts (Fig. 2a, Additional file 2: Table S2). Among beef cuts, we detected the largest (*n* = 30) RSGs in the longissimus lumborum cut, followed by 19 RSGs in the neck cut (Fig. 2a). The functional annotation and pathway enrichment of RSGs indicated the meat quality characteristics of the beef cut. For instance, the chuck-related RSGs were significantly enriched in 2-oxocarboxylic acid metabolism (*P* value = 1.00e-02) and biosynthesis of amino acids (*P* value = 3.70e-02), the longissimus lumborum-related RSGs for ribosome (*P* value = 3.09e-03) and proteasome complex (*P* value = 4.43e-02), the tenderloin-related RSGs for calcium ion binding (*P* value = 3.32e-03) and actin cytoskeleton (*P* value = 1.02e-02) (Fig. 2b, Additional file 3: Table S3).

**Fig. 3:**
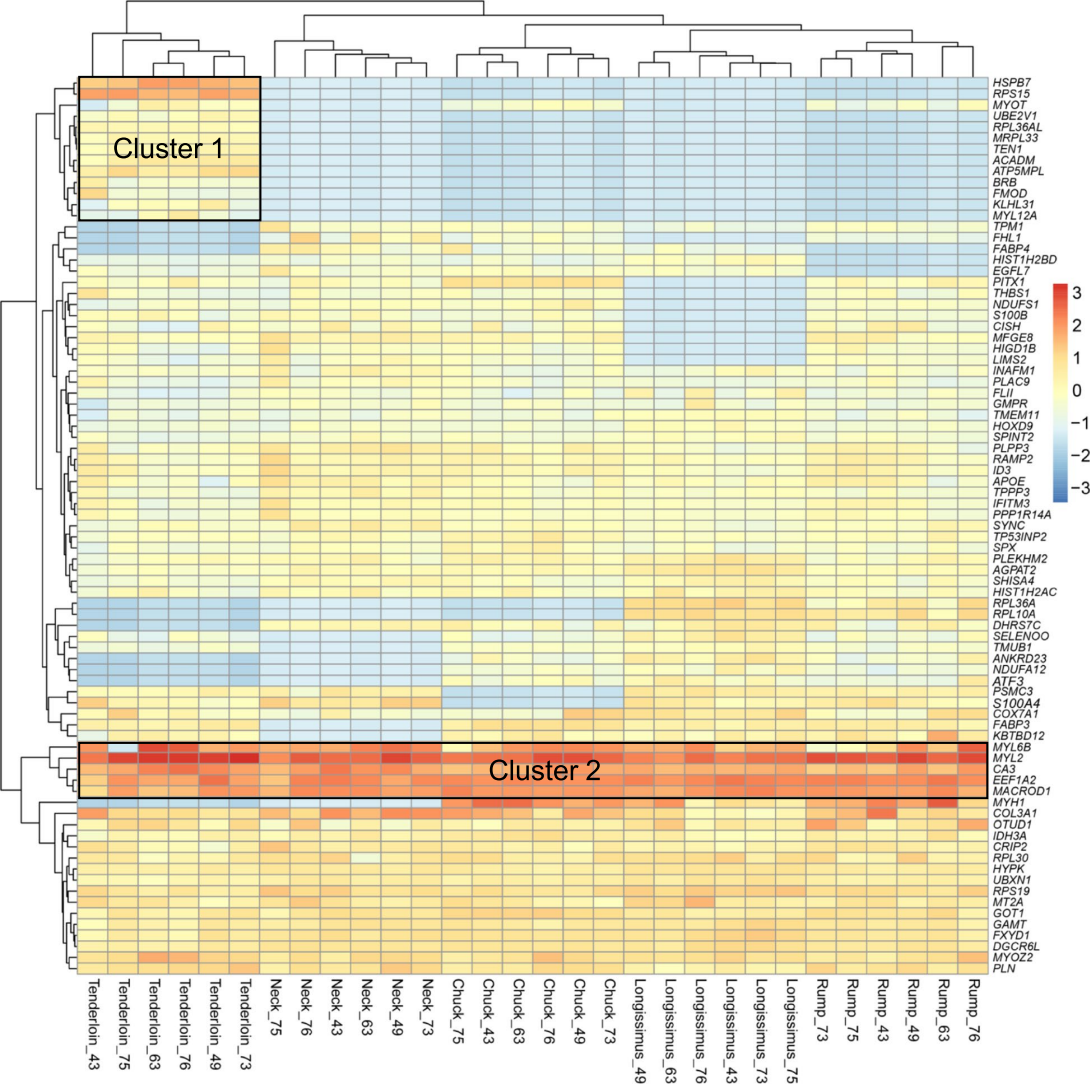
Clustering of expression patterns of 80 RSGs. Color intensity represents expression level estimated through log_10_ normalized FPKM, red represents highly expressed, and blue represents lowly expressed. The x-axis represents beef cuts (*n* = 6), namely the tenderloin, neck, chuck, longissimus lumborum and rump; the y-axis represents RSGs expression levels

To identify master regulators of RSGs involved in biological processes, we performed TF analysis on five types of beef cuts. In the chuck cut, a total of four TFs were identified including ESRRA (NES = 8.317), RXRA (NES = 4.130), RCOR1 (NES = 3.422), and ESR1 (NES = 3.153) (Fig. 2c, Additional file 4: Table S4). In the tenderloin and neck cut, six TFs were identified, respectively, namely TEAD4, POLR2A, TAF1, JUN, FOSL, and MAFK, as well as SIN3A, BCL3, JUND, CTCF, TEAD4, and EP300 (Fig. 2d, e). The TFs identified in other beef cuts were shown in Additional file 5: Fig. S1. Meanwhile, we investigated the PPI network using our list of RSGs in different beef cuts, the sub-networks revealed that the major clusters consist of a large number of RSGs in beef cuts. We found a strikingly consistent pattern among RSGs. Severn RSGs associated with ribosome function showed a high connection in sub-network, while four out of nine muscle fiber structure genes displayed a similar pattern (Additional file 5: Fig. S2). In addition, we performed an RSG expression pattern profile and observed that a cluster of RSGs was highly expressed in the tenderloin cut. Meanwhile, the hierarchical cluster analysis found the tenderloin cut was separated from other beef cuts, and the RSGs in cluster 1 were highly expressed in the tenderloin cut, indicating the differences among them (Fig. 3). We also observed that RSGs in cluster 2 have high expression levels than other RSGs (Fig. 3).

### Region-specific modules analysis

**Fig. 4:**
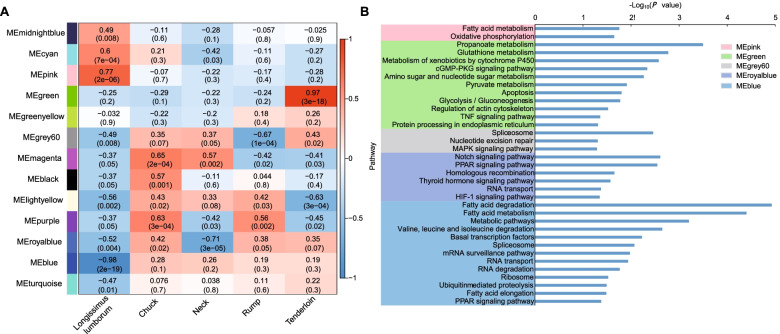
Region-specific modules detected. **a** Heatmap between 13 modules and five types of beef cuts. Boxes contain Pearson correlation coefficients and their associated *P* values. The red color indicates that the given beef cut has a strong positive correlation relative to other beef cuts. The blue color indicates that the given beef cut has a strong negative correlation relative to other beef cuts. **b** Pathway enrichment of region-specific module genes. The x-axis represents the significance of the pathway term, expressed as -log_10_ (*P*-value), and the y-axis represents the pathway term

To explore the associations between gene expression and meat quality, we performed co-expression analysis on the expression levels of genes in five types of beef cuts. Using a WGCNA approach, we divided the filtered 4511 genes into 13 co-expressed gene modules. To obtain region-specific modules, we assessed the association between 13 modules and five types of beef cuts. Under the criteria of the correlation coefficient (*r* > 0.60) and *P*-value (*P* < 1.0e-2), we identified seven region-specific modules in our analysis (Fig. 4a, Additional file 6: Table S5). For instance, the MEpink module (*r* = 0.77, *P*-value = 2.00e-6) was positively correlated with longissimus lumborum, the MEblue module (*r* = − 0.98, *P*-value = 2.00e-19) was negatively correlated with longissimus lumborum. (Fig. 4a). The MEmagenta module was significantly correlated with chuck (*r* = 0.65, *P*-value = 2.00e-04) and neck (*r* = 0.57, *P*-value = 2.00e-3), respectively. The MEgreen module (*r* = 0.97, *P*-value = 3.00e-18) and MElightyellow module (*r* = − 0.63, *P*-value = 3.00e-4) were closely related to tenderloin (Fig. 4a).

**Fig. 5:**
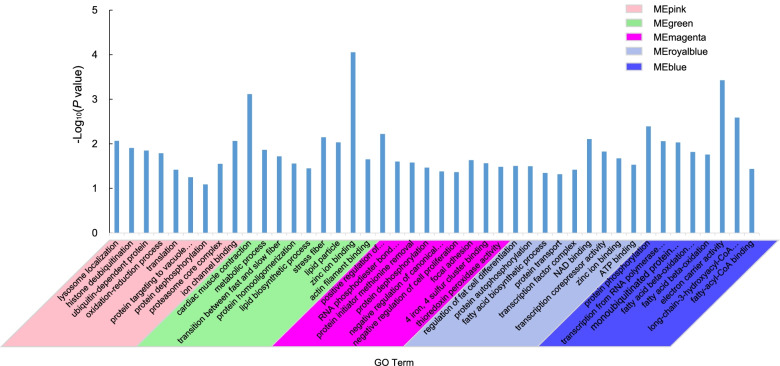
Function annotation of region-specific module genes. The x-axis represents the GO term, and the y-axis represents the significance of the GO term, expressed as -log_10_ (*P*-value)

Pathway enrichment analysis showed that genes included in the MEpink module were involved in fatty acid metabolism (bta01212) and oxidative phosphorylation (bta00190). The genes included in the MEblue module were mainly involved in valine, leucine, and isoleucine degradation (bta00280) and fatty acid elongation (bta00062) (Fig. 4b). The functional annotations of genes in region-specific modules indicated that the MEpink module was mainly involved in proteasome core complex (GO:0005839), ion channel binding (GO:0044325) and oxidation-reduction process (GO:0055114). The MEblue module was mainly involved in mRNA processing (GO:0006397), protein phosphorylation (GO:0006468), and fatty acid beta-oxidation (GO:0006635). The genes within the MEmagenta module were involved in positive regulation of protein catabolic process (GO:0045732), 4 iron, 4 sulfur cluster binding (GO:0051539) and thioredoxin peroxidase activity (GO:0008379) (Fig. 5). In addition, the MEgreen module was mainly involved in the transition between fast and slow fiber (GO:0014883), actin filament binding (GO:0051015) and lipid biosynthetic process (GO:0008610). The MEroyalblue module was related to the neck cut and mainly involved in protein autophosphorylation (GO:0046777), protein transport (GO:0015031) and fatty acid biosynthetic process (GO:0006633) (Fig. 5).

### Candidate genes for meat quality among beef cuts

**Fig. 6:**
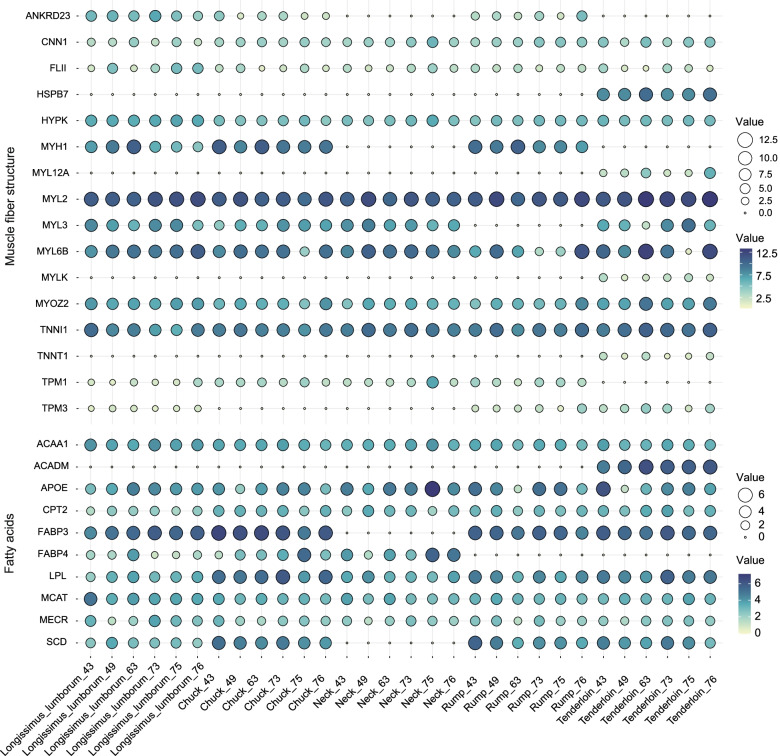
Muscle fiber structure and fatty acid candidate gene expression profile. The x-axis represents five types of beef cuts (*n* = 6), namely the longissimus lumborum, chuck, neck, rump, and tenderloin. The y-axis represents the expression level of candidate genes. The size of the circle and the intensity of the color indicate the degree of the expression level of candidate genes, and the value represents log_2_ (FPKM) normalization

To detect the genes that affect the differences in meat quality among different beef cuts, we identified 91 candidate genes related to meat quality traits from RSGs and co-expression genes (Additional file 7: Table S6). These candidate genes were mainly involved in muscle fiber structure, fatty acids, amino acids, protein processing, energy production and conversion biological processes. For instance, 16 and 10 candidate genes were identified in muscle fiber structure and fatty acids, respectively. The gene expression patterns indicated that the expression levels of candidate genes were diverse among different beef cuts. Four genes (*HSPB7*, *MYL12A*, *TNNT1*, *MYLK*) were up-regulated in tenderloin cut compared with other beef cuts in terms of muscle fiber structure (Fig. 6, Additional file 7: Table S6). In fatty acids, we also observed that the *ACADM* gene was uniquely expressed in tenderloin cut. Three (*ACADS*, *ALDH2*, *IVD*) and six genes (CRIP2, *CNBP*, *LIMS2*, *PHPT1*, *SNTA1*, *SUMO1*) were involved in amino acids and ion channel binding, respectively (Additional file 5: Fig. S3, Additional file 7: Table S6). A total of 17 genes were involved in protein processing, of which seven genes belong to RSGs, (Additional file 5: Fig. S4, Additional file 7: Table S6). In addition, we found 39 genes (e.g., *ATP8*, *COX8B*, *NDUFB6*) were involved in energy production and conversion. These genes show significantly different expressions among beef cuts (Additional file 5: Fig. S5, Additional file 7: Table S6).

### Validation of the candidate genes using RT-qPCR

To validate the expression accuracy of the candidate genes derived from RNA-seq, we performed RT-qPCR for five types of beef cuts from the same individual samples. Four candidate genes including *ALDH2*, *CANX*, *IVD*, *PHPT1* were selected for the subsequent analysis. Consistent with gene expression changes from RNA-seq analysis, we found significant differences among the gene expression values among five types of beef cuts (Additional file 5: Fig. S6). There were significant differences in the expression of candidate genes between tenderloin and chuck, for instance, *ALDH2* (*P* = 0.02996), *CANX* (*P* = 0.0375). *IVD* gene showed different expressions between tenderloin and rump. Meanwhile, significant differences were observed between longissimus lumborum and chuck for *ALDH2* (*P* = 0.00516), *PHPT1* (*P* = 5.48e-06). Our RT-qPCR analyses confirmed that candidate genes for meat quality were differentially expressed across beef cuts and further supported RNA-seq results.

## Discussion

We performed a comparative analysis of the expression pattern using high-throughput sequencing in different muscle tissues from various beef cuts. Many previous studies have been applied to investigate the genetic basis of meat quality using different gene expression and co-expression analyses [14–16]. Our study systematically examined the associations between gene expression and meat quality, and identified region-specific modules. Our findings provide valuable insights into understanding the expression content of candidate genes affecting meat quality among beef cuts, which may further shed light on the region-specific expression patterns in farm animals.

Investigating region-specific gene expression can help to understand life processes and physiological functions. The region-specific transcriptomic analyses have been reported in many farm animals, for instance, water buffalo [17], sheep [18], pigs [19], cattle [20], which provide valuable insights into understanding the expression regulation involved with functional differences among regions. Our study identified a total of 80 RSGs in multiple beef cuts from different regions based on the previously described method [13]. The expression pattern of RSGs showed that the RSGs in cluster 1 were highly expressed in tenderloin compared to other beef cuts. Among them, *HSPB7* and *RPS15* have significantly high expression in tenderloin cut, which may contribute potential function for physiological differences in meat quality. Although the five RSGs in cluster 2 have higher expression levels than other RSGs, *MYL6B*, *CA3* and *MYL2* showed region-specific expression in the tenderloin cut, and *EEF1A2* and *MACROD1* showed region-specific expression in the longissimus lumborum cut (Additional file 2: Table S2), which may affect meat tenderness [21].

To explore the system-level functionality of genes for muscle-specific molecular differences, we applied a co-expression network approach to identify region-specific modules and within-module candidate genes associated with meat quality. These modules were enriched in a number of pathways (e.g., fatty acid metabolism, oxidative phosphorylation and protein catabolic process), suggesting their complex mechanisms underlying meat quality. By analyzing the co-expression genes within these modules, we identified a series of hub genes (e.g., *TNNT1*, *ACADM*, *SCD*) related to the nutritional components of meat. These hub genes have been reported in many domestic animals, such as in sheep [22], pigs [23], cattle [24].

The beef quality grade of different beef cuts can be affected by multiple physiological processes. Gene expression changes were widespread across different beef cuts, and identification of candidate genes may contribute to understanding the expression regulation of the meat quality. Notably, we identified 91 candidate genes using both region-specific gene expression and co-expression network approaches, and these genes were mainly enriched in muscle fiber structure, fatty acids, amino acids, ion channel binding, protein processing, energy production and conversion. A previous study found that the muscle type greatly influenced the meat quality and sensory property between tenderloin and longissimus lumborum [25]. In this study, we detected two genes (*TNNI1* and *TNNT1*) with higher expressions in the tenderloin and longissimus lumborum, which may suggest that these genes were associated with drip loss and meat color, respectively [26]. *MYL2* gene showed region-specific expression in the tenderloin, and the differential phosphorylation level of MYL2 protein may be the crucial factor in regulating muscle rigor mortis [27]. *CNN1* gene was down-regulated in longissimus lumborum and chuck as compared to rump and tenderloin and was specifically expressed in smooth muscle cells and had a fine-tuning effect on smooth muscle contraction [28]. The protein levels of TPM1 and TPM3 may regulate marbling development and improvement of meat quality grades in cattle [29].

As for fatty acids processing, three genes (*MCAT*, *MECR, ACAA1*) were identified with higher expression in longissimus lumborum as compared with those of other beef cuts. The *SCD* gene encoded key enzymes for lipogenesis, which regulated and catalyzed the conversion of saturated fatty acids into monounsaturated fatty acids [30], and was associated with meat tenderness [31]. The tenderloin has the potential to be beneficial for human health due to it has high content of polyunsaturated fatty acids, especially essential fatty acids [32]. Moreover, we detected three genes for amino acid change. *ALDH2* gene is localized in mitochondria, and encodes a protein with an amino acid change from glutamate to lysine [33], which was up-regulated in longissimus lumborum. Also, IVD was observed that strongly correlated with tenderness and intramuscular fat [34], which show higher expression in the tenderloin and rump muscle (Additional file 7: Table S6). These two genes were reported that play a key role in regulating energy homeostasis and nutritional metabolism of humans and animals [35]. In addition, we identified several genes involved with ion channel binding, protein processing, and energy production and conversion*.* We observed *SNTA1* genes exhibited a more than 1.4-fold increase in expression in longissimus lumborum. This finding was consistent with that a high SNTA1 protein level was detected in double-muscled cattle, suggesting *SNTA1* mainly regulates the influx of calcium in skeletal muscle to maintain muscle activity [36]. In particular, *GAPDH* and *RNF181* exhibited more than a 1-fold increase in almost all beef cuts, which were reported that related to beef tenderness [37]. Meanwhile, two genes (*CANX* and *SMAD5*) were detected with significantly lower expression in longissimus lumborum (Additional file 7: Table S6).

Overall, the identified candidate genes for beef quality contribute to our understanding of their transcriptional regulation in different beef cuts. Furthermore, recent advances in single-cell RNA-sequencing technology have enabled transcriptional profiles to be measured at a single-cell resolution [38]. Integrative analyses of single-cell mRNA data with multi-omics data can provide more comprehensive insights into the molecular mechanism of meat quality and cell type-specific gene regulation than single-cell mono-omics analysis [39].

## Conclusions

In this study, we identified 80 RSGs from five types of beef cuts and obtained seven region-specific modules using RNA sequencing. Our findings revealed 91 candidate genes related to meat quality, including 29 RSGs. Functional annotations suggested that these candidate genes were mainly involved in muscle fiber structure, fatty acids, amino acids, ion channel binding, protein processing, energy production and conversion. Our results provided valuable insights into understanding the transcriptomic regulation of meat quality in beef cattle.

## Methods

### Sample collection

Six male Chinese Simmental beef cattle from different sires and dams were collected from Inner Mongolia Oaks Co., Ltd. in Ulgai, Xilingol League, Inner Mongolia of China. Before slaughtering, these cattle were fattened under the same feeding and management conditions (Jingxin Xufa Agricultural Development Co., Ltd., Hebei) until they were 2 years old with a weight of ~ 700 kg. Then, these cattle were transferred to Inner Mongolia Zhongao Food Co., Ltd. for slaughter. Tissue samples were collected with the approval of the Science Research Department of the Institute of Animal Science, Chinese Academy of Agricultural Sciences under IAS2020–48. A total of five types of beef cuts, including chuck, neck, rump, tenderloin and longissimus lumborum were collected and saved in RNAlater (Qiagen) and snap-frozen in liquid nitrogen.

### RNA extraction

Total RNA was extracted from each sample using the Trizol method and subjected to quality control by the NanoDrop® 2000 (Thermo, CA, USA) and treated with DNase I (RNase-free) following the manufacturer’s instructions. Then RNA quality was determined by the Agilent Bioanalyzer 2100 system (Agilent Technologies, CA, USA).

### Library preparation and sequencing

mRNA libraries were prepared following the TruSeq Stranded library protocols using 5 μg of total RNA. The 250–300 bp fragment size was selected with AMPure XP beads and used for PCR enrichment of the fragment for library construction. To ensure the quality of the library, PCR products were purified (AMPure XP beads) and quality was assessed on the Agilent Bioanalyzer 2100 system. Finally, raw sequence data were generated per sample using the Illumina Nova Seq 6000 system.

### Data quality control and mapping

The raw paired-end data were trimmed for high-quality reads using FASTP software with default parameters [40]. Then, clean data for each sample were mapped to the reference genome (ARS-UCD1.2, https://asia.ensembl.org/Bos_taurus/Info/Index) using HISAT2 [41]. The transcripts and expressed genes were assembled and quantified by STRINGTIE (v.2.14) [12]. The expression levels of transcripts and genes were estimated using read counts and fragments per kilobase of transcript per million mapped reads (FPKM), respectively.

### Region-specific genes detection

To identify RSGs, we used the methods as previously described [13]. In brief, RSGs were defined according to three criteria: 1) The FPKM value of the candidate gene in one type of beef cut was more than three times that of others; 2) The FPKM value of candidate gene in one type of beef cut was greater than 50% of the average expression level in others; 3) The expression level of candidate genes was at the top 25% of all genes in each beef cut. The hierarchical clustering of RSGs was displayed using the Pheatmap software package. Functional annotation and enrichment analysis of RSGs using the Database for Annotation, Visualization and Integrated Discovery (DAVID) v.6.8 with Bonferroni’s multiple test [42]. Meanwhile, RSGs in five types of beef cuts were used to predict TFs using iRegulon tool (v.1.3). The regulation network of TFs and RSGs was constructed using Cytoscape software (v.3.7.1) [43]. The size of TFs was displayed based on their NES (Normalized enrichment score). Moreover, the protein-protein interaction (PPI) network of 81 RSGs in different beef cuts was generated using the String database (https://string-db.org/), and the networks were visualized using Cytoscape (v.3.7.1).

### Identification of region-specific modules

The gene network analysis was performed using a weighted gene correlation network analysis (WGCNA) [44]. Briefly, we firstly constructed an expression matrix of 4511 genes (FPKM > 1) using 30 samples. The soft threshold (β = 5) was determined based on the principle of scale-free distribution. Principal component analysis was performed on the expression matrix of genes in each module to obtain module eigengene (ME). To identify the region-specific modules, we constructed a design matrix X, where each row corresponded to a sample, and each column corresponded to a tissue with the same tissue was 1, and the non-identical tissue was 0. The correlation coefficient between the matrix X and the module ME was further calculated with the Pearson correlation coefficient. The module with a correlation coefficient larger than 0.60 and *P*-value less than 1.0e-2 was considered as a region-specific module. We performed functional enrichment analyses for region-specific module genes based on the DAVID database [42].

### Real-time quantitative PCR (RT-qPCR) analysis

To validate the expression accuracy of the candidate genes for meat quality, four candidate genes were selected for qRT-PCR analyses using the QuantStudio 7 Flex real-time PCR System (Life Technologies, Carlsbad, CA, USA). Total RNA from the tenderloin, longissimus lumborum, rump, neck, chuck samples were extracted using Trizol reagent (Invitrogen, New York, NY, USA), and quality was checked by NanoDrop® 2000 (Thermo, CA, USA) and treated with DNase I (RNase-free). RNA was then reverse transcribed to cDNA using the Prime Script™ RT Reagent kit with gDNA Eraser (Takara, Dalian, China). Primers for the candidate and the housekeeping gene (*GAPDH*) were designed using the Primer Premier 5.0 software (Additional file 8: Table S7) and synthesized by Sangon Biotech (Sangon, Shanghai, China). The amplification cycle involved an initial denaturation step at 95 °C for 3 min, followed by 40 cycles at 95 °C for 2 s, 60 °C for 20 s. The 2^−ΔΔCt^ method was used to transform Ct values. The expression levels of five beef cuts were compared with the basal value using the nonparametric Kruskal-Wallis and Nemenyi test.

## Supplementary Information

**Additional file 1:** **Table S1. **Reads mapping summary of chuck, neck, rump, tenderloin and longissimus lumborum.

**Additional file 2:** **Table S2. **Identification of region-specific genes (RSGs).

**Additional file 3:** **Table S3. **Functional annotation and enrichment analysis of RSGs.

**Additional file 4:** **Table S4.** RSGs transcription factor analysis.

**Additional file 5:** **Figure S1. **TF analysis of RSGs in rump and longissimus lumborum cuts. **Figure S2. **The PPI network of RSGs.** Figure S3. **Amino acids and ion channel binding candidate gene expression profile.** Figure S4. **Protein processing candidate gene expression profile. **Figure S5.** Energy production and conversion candidate gene expression profile. **Figure S6.** Validation of the expression levels of candidate genes related to meat quality traits using RT-qPCR.

**Additional file 6:** **Table S5.** The identified region-specific modules.

**Additional file 7:** **Table S6.** The summarize of candidate genes related to meat quality traits in longissimus lumborum, chuck, neck, rump and tenderloin beef cuts.

**Additional file 8:** **Table S7.** RT-qPCR primer sequences for candidate genes related to meat quality traits.

**Additional file 9. **Author checklist.

## Data Availability

The datasets used and analyzed during the current study are available from the corresponding author on academic request.

## Abbreviations

RSGs: Region-specific genes
TFs: Transcription factors
FPKM: Fragments per kilobase of transcript per million mapped reads
NES: Normalized enrichment score
WGCNA: Weighted gene correlation network analysis
ME: Module eigengene
DAVID: Database for Annotation, Visualization and Integrated Discovery
