# Supplementary material for: Comparative transcriptomic analysis reveals region-specific expression patterns in different beef cuts
Source: BMC Genomics. 2022 May 20;23:387. doi: 10.1186/s12864-022-08527-3 (PMC9123670; doi:10.1186/s12864-022-08527-3)
Supplement: Supplementary file 5 — Additional file 5: Figure S1. TF analysis of RSGs in rump and longissimus lumborum cuts. Figure S2. The PPI network of RSGs. Figure S3. Amino acids and ion channel binding candidate gene expression profile. Figure S4. Protein processing candidate gene expression profile. Figure S5. Energy production and conversion candidate gene expression profile. Figure S6. Validation of the expression levels of candidate genes related to meat quality traits using RT-qPCR. [file 12864_2022_8527_MOESM5_ESM.pdf]

Supplementary Figures

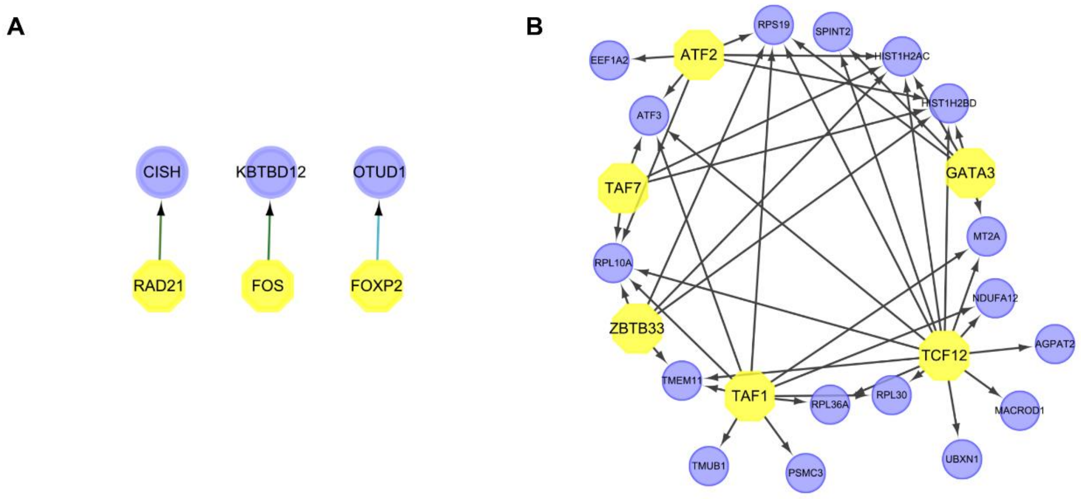

Figure S1. TF analysis of RSGs in rump and longissimus lumborum cuts. a. TF analysis of RSGs in rump cut. Yellow nodes represent TFs and blue nodes represent regulatory target genes. **b**. TF analysis of RSGs in longissimus lumborum cut. Yellow nodes represent TFs and blue nodes represent regulatory target genes.

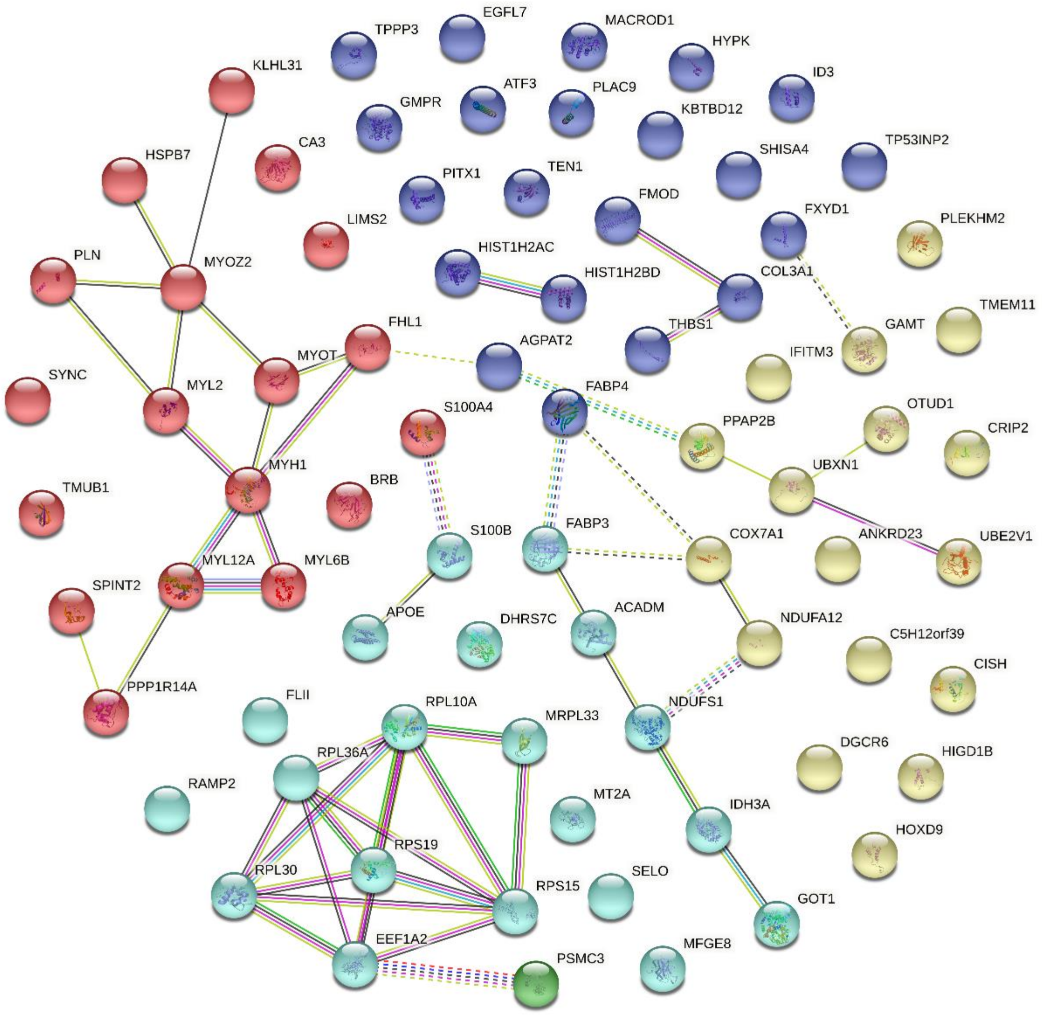

Figure S2. The PPI network of RSGs. Node represents genes and edge connects the nodes to indicate interactions among them.

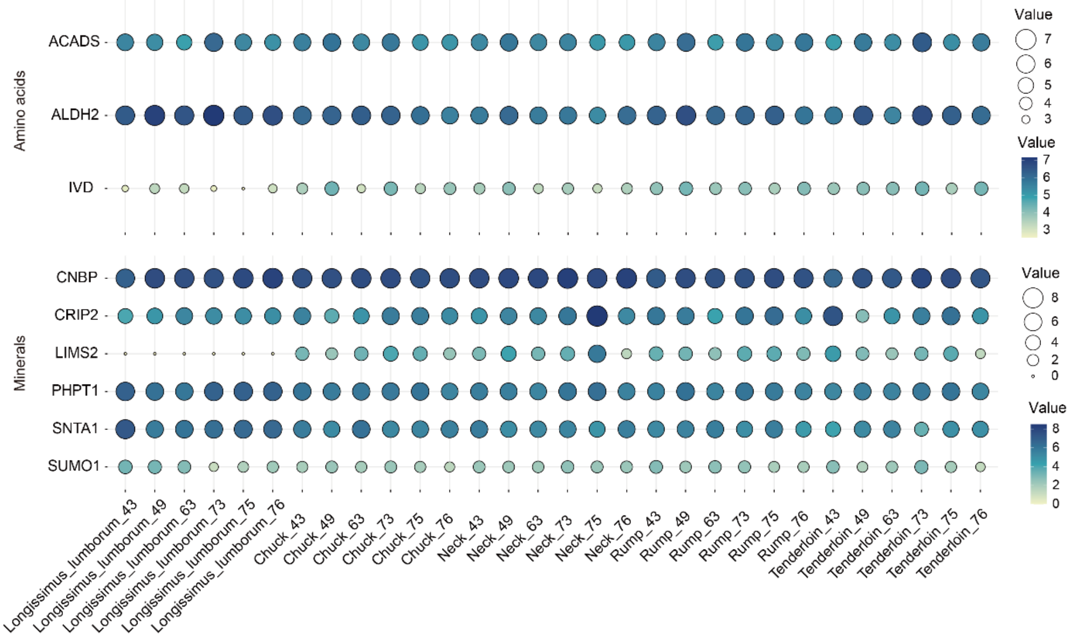

Figure S3. Amino acids and ion channel binding candidate gene expression profile. The x-axis represents five types of beef cuts (n=6), namely the longissimus lumborum, chuck, neck, rump and tenderloin. The y-axis represents the expression level of candidate genes. The size of the circle and the intensity of the color indicate the degree of the expression level of candidate genes, and the value represents $\log_2$ (FPKM)) normalization.

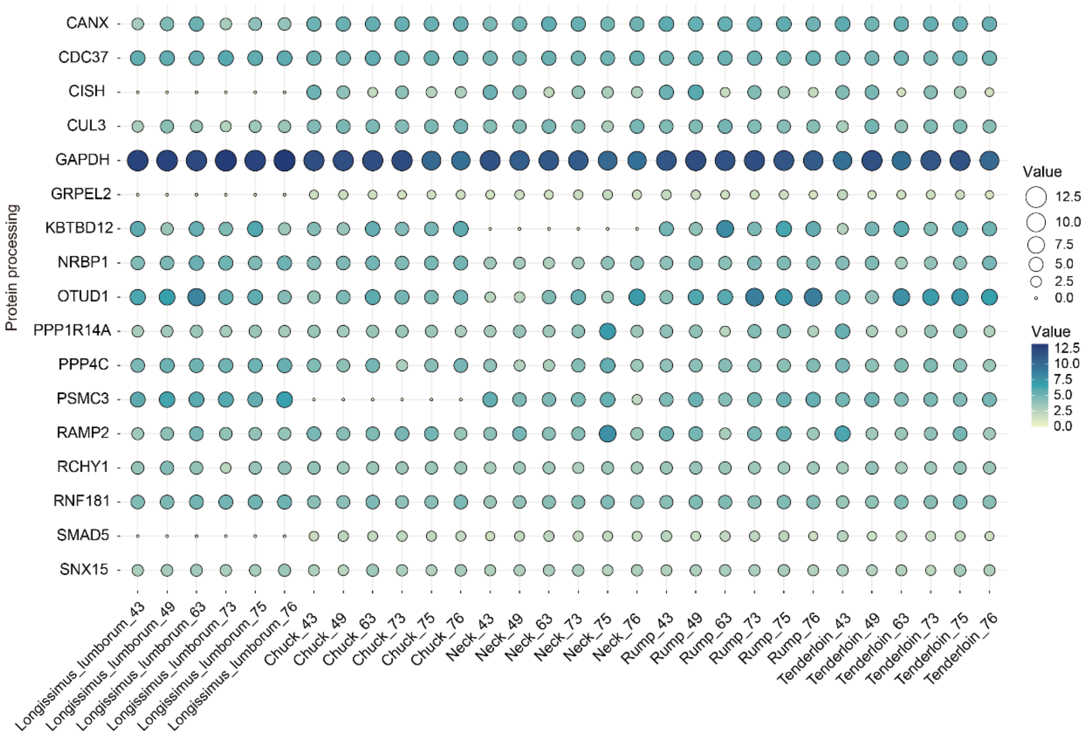

Figure S4. Protein processing candidate gene expression profile. The x-axis represents five types of beef cuts (n=6), namely the longissimus lumborum, chuck, neck, rump and tenderloin. The y-axis represents the expression level of candidate genes. The size of the circle and the intensity of the color indicate the degree of the expression level of candidate genes, and the value represents log_2 (FPKM)) normalization.

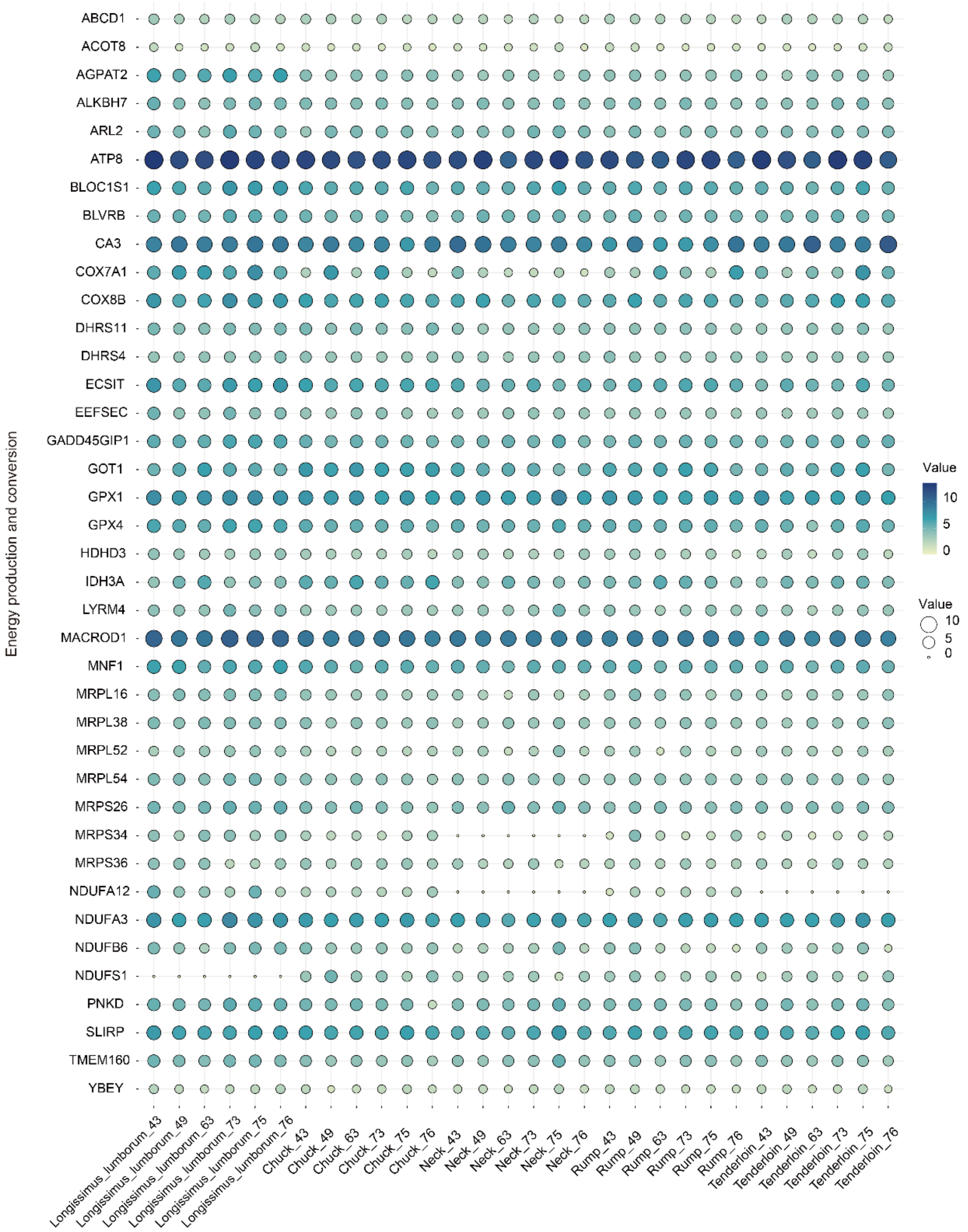

Figure S5. Energy production and conversion candidate gene expression profile. The x-axis represents five types of beef cuts (n=6), namely the longissimus lumborum, chuck, neck, rump and tenderloin. The y-axis represents the expression level of candidate genes. The size of the circle and the intensity of the color indicate the degree of the expression level of candidate genes, and the value represents $\log_2$ (FPKM)) normalization.

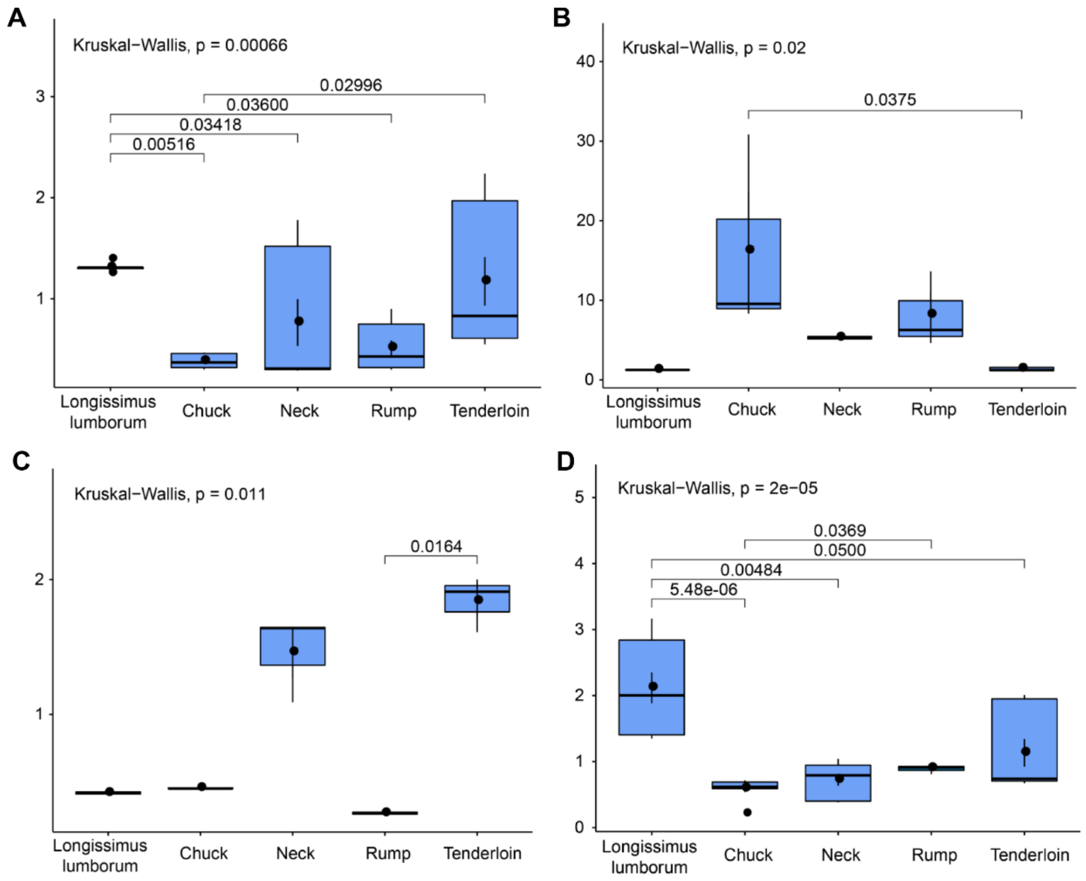

Figure S6. Validation of the expression levels of candidate genes related to meat quality traits using RT-qPCR. Boxplots show the mRNA expression levels of four candidate genes randomly selected in five types of beef cuts in Chinese Simmental beef cattle (n = 6). (A) *ALDH2*, (B) *CANX*, (C) *IVD*, (D) *PHPT1*. The ordinate is the relative expression level value, and the abscissa is the name of the bovine tissue sample. In the box plot: the maximum value (top of the line), the minimum value (low end of the line), the median (black point), the upper quartile (the upper border of the rectangle), the lower quartile (the bottom border of the rectangle), and the invalid data (out-of-line outliers).
